# Entangled stressors: a review on the interactions between microplastics and climate change in aquatic ecosystems

**DOI:** 10.3389/ftox.2026.1833046

**Published:** 2026-05-18

**Authors:** Jeffrey Lebepe, Nana MD Buthelezi, Madira C. Manganyi

**Affiliations:** Department of Biology and Environmental Science, Sefako Makgatho Health Sciences University, Pretoria, South Africa

**Keywords:** aquatic ecosystem, climate change, microplastic pollution, microplastic toxicity, temperature

## Abstract

Microplastics (MPs) have become a cause for concern in aquatic ecosystems due to their potential to cause toxicity, which can be enhanced when co-occurring with other stressors. The contribution of climate change to MP toxicity mechanisms has been shown to be diverse, with more questions still remaining unanswered. Climate change influences the hydrologic dynamics of the aquatic environment through floods, which result in an increased abundance of MPs, whereas increased temperature in the aquatic ecosystem accelerates the leaching of additives from both macro and MPs. Moreover, increased metabolism as a result of increased temperature may also accelerate the uptake of MPs as they mistake them for food. While the combined effects of thermal stress and MP exposure pose serious threats to aquatic life, some organisms may exhibit resilience under certain conditions or adaptive mechanisms that could mitigate the impacts of MPs and associated contaminants. More studies are recommended to understand the long-term implications of these stressors in aquatic ecosystems and the potential for recovery after system restoration. Climate change and MP pollution are global phenomena; therefore, addressing their threats to aquatic ecosystems requires global collaborative efforts with regard to policy direction and the involvement of citizen science to integrate end-users in solution seeking. This review consolidates existing information on MP pollution and the role of climate change on the dynamics, fate, and toxicity effects of microplastics. Moreover, the review identifies some research gaps on the mechanisms driving their interactions, effect pathways in aquatic ecosystems, and the possible policy direction to enhance strategies to address this global threat.

## 1 Introduction

Microplastics (MPs) have become a cause for concern as they are widely detected across multiple environmental matrices and their potential toxicity, particularly in aquatic environments (Singh et al., 2025). Numerous anthropogenic activities, including wastewater treatment works, industrial activities, urbanization, and agricultural practices, have been identified as potential drivers of MP pollution (Wang et al., 2017; Prasath et al., 2025). Microplastic concentrations have recently shown a significant increase in freshwater ecosystems, which has stimulated research interest in their effects on the functioning and resilience of these systems (Alfaro-Núñez et al., 2021; Alfonso et al., 2021; Ai et al., 2024). Nevertheless, stressors rarely act solely in ecosystems, and studies have shown that microplastics interact with chemical pollutants, resulting in synergistic effects (Wang et al., 2021; Ai et al., 2024). Another stressor that exhibits a clear interaction with MPs is climate change, which has been shown to have both direct and indirect association pathways (Haque and Fan, 2023; Zheng et al., 2025).

With the Earth becoming warmer due to climate change, the fate of MPs and their effect dynamics are receiving increasing attention due to uncertainties around their interaction. For the direct association, evidence showed that varying temperatures may change MP effects and effect pathways in aquatic biota (Chang et al., 2022; Klasios et al., 2024). In freshwater environments, plastics leach additives, with the process increasing for MPs due to their high surface area. Moreover, increasing temperature was found to accelerate the MP additive leaching, resulting in a pH drop, hence, acidification (Huang et al., 2022). According to Romera-Castillo et al. (2023) and Lawrence and Roy (2021), plastic degradation through solar radiation may result in high organic acids and the emission of carbon dioxide (CO_2_), through an increased release of dissolved organic carbon. This concept changes the whole dynamics of climate change and MP interaction, as acidification influences the toxicity of other contaminants, such as metals (Feng et al., 2017; Ali et al., 2019). Nevertheless, climate change may increase the rate of MP degradation and dispersal due to high fragmentation through increased temperature (Zheng et al., 2025). For the indirect association between climate change and MP pollution, hydrological extremes wash plastics into the rivers, where the ultraviolet radiation accelerates the degradation (Haque and Fan, 2023).

There is considerable evidence linking climate change to the occurrence and effects of MPs; however, some knowledge gaps hinder a comprehensive understanding of the association between these two stressors and their effect pathways. This review consolidates existing information on PM pollution and the role of climate change on the dynamics, fate, and toxicity effects of microplastics. Moreover, the review identifies some research gaps on the mechanisms driving their interaction and effect pathways in aquatic ecosystems.

## 2 Climate change effects on microplastic fate, transport and toxicity

**FIGURE 1:**
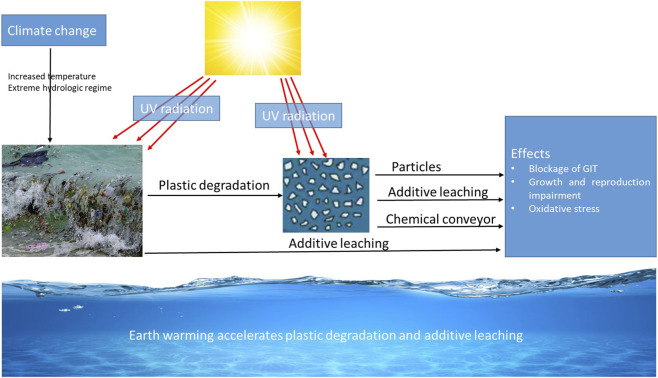
Microplastics association with climate change and their effect pathways.

Climate change is increasingly recognized as a critical factor influencing the environmental fate and transport of MPs by driving their degradation and distribution across ecosystems. Moreover, it influences the hydrologic dynamics of aquatic ecosystems, whereas the UV radiation may accelerate particle degradation processes, altering the persistence and bioavailability of plastic particles, resulting in synergistic toxicity effects (Figure 1). Altered hydrological regimes, driven by shifts in precipitation patterns and extreme weather events, are key determinants of MP transport (Zhang et al., 2023). Extreme weather events, including floods, droughts, and storm surges, act as episodic drivers of plastic mobilization and redistribution (Figure 1). Floods can entrain large volumes of macroplastics and previously settled MPs from riverine and urban landscapes into aquatic ecosystems, dramatically increasing downstream fluxes (Martuscelli, 2025). Conversely, droughts reduce water flow, concentrating MPs in residual water bodies and sediments, potentially intensifying localized ecological impacts (Chen et al., 2020). Storm-driven events, including hurricanes and typhoons, not only resuspend sequestered plastics from sediments but also transport them across coastal and marine environments, effectively linking terrestrial, freshwater, and marine microplastic reservoirs (Nafea et al., 2025). According to Belioka and Achilias (2024), hydrological extremes also influence sedimentation and resuspension cycles, modulating the long-term retention and lateral redistribution of MPs across watersheds.

Changes in the cryosphere and rising sea levels further contribute to the redistribution of plastic debris. Melting glaciers and ice sheets release long-trapped microplastics into downstream rivers and coastal systems, introducing contaminants into previously pristine environments. Seasonal ice melt and permafrost thawing can mobilize both synthetic polymers and microplastic-laden sediments, altering freshwater and estuarine transport pathways (Gaylarde et al., 2023). Additionally, sea-level rise modifies coastal hydrodynamics, enhancing sediment erosion, resuspension, and the lateral movement of microplastics. These processes collectively facilitate the long-range dispersal of plastics, linking polar, temperate, and tropical ecosystems and challenging traditional assumptions of MPs accumulation hotspots (Bergmann et al., 2017).

Temperature increases associated with global warming accelerate the physical and chemical degradation of polymers, altering MP fragmentation rates (Song et al., 2022). Thermal stress enhances photochemical oxidation and hydrolytic cleavage of polymer chains, resulting in surface embrittlement and fragmentation into micro- and nanoscale particles (Song et al., 2022; Kumar, 2023; Song et al., 2023). Temperature-dependent changes in polymer density and buoyancy also modify particle transport in aquatic systems, impacting residence time and spatial distribution patterns (Lusher, 2015). At a nanoscale, particle dispersal increases, resulting in long-range transport (Jansen et al., 2024). Moreover, nanoplastics (<1 µm) have a large surface area, hence, facilitating uptake and remediation of chemical pollutants by providing adequate space for adsorption (Chen et al., 2024). Additionally, the high surface area of nanoparticles may provide for microbial biofilms, which can enhance biodegradation efficiency (Kumar et al., 2026). On the other hand, nanoplastics produce greenhouse gases such as methane and ethylene when exposed to solar radiation as a result of gas exchange and CO_2_ circulation (Kakar et al., 2023). Besides accelerating degradation, an increase in temperature may result in the loss of ice cover, releasing trapped MPs and enhancing their resuspension and redistribution in aquatic ecosystems (Gaylarde et al., 2023). These processes facilitate long-range dispersal, linking polar, temperate, and tropical systems and challenging conventional assumptions regarding microplastic hotspots and sinks (Martina and Castelli, 2023).

Another climate change related factor driving fate, transport and toxicity of MPs is the ultraviolet (UV) radiation that has the capacity to enhance degradation and additives leaching (Jansen et al., 2024). The UV radiation intensified under climate change scenarios due to ozone layer fluctuations and increased solar exposure, drives photochemical aging of MPs (Figure 1). The UV-induced oxidation generates surface functional groups that increase hydrophilicity, alter sorption capacity, and accelerate fragmentation (Barnes et al., 2022). These photochemically weathered particles are more prone to mechanical breakdown and enhanced environmental mobility, potentially increasing bioavailability to aquatic organisms. The synergistic effects of UV radiation and temperature amplify degradation rates and influence long-term polymer persistence in surface waters and sediments (Zhu et al., 2020).

The overall climate-driven alterations in hydrology, temperature, and UV exposure collectively determine the fate and transport of microplastics. Moreover, temperature-dependent degradation, episodic transport from extreme events, and cryospheric contributions collectively dictate the spatial and temporal distribution of MPs. The interplay between climate-driven factors and MP dynamics highlights the need to integrate environmental change projections into pollution monitoring and mitigation frameworks. Therefore, understanding their interactive mechanisms is critical for predicting environmental exposure, assessing potential ecological risk, designing targeted remediation strategies, developing policies and informing management strategies that integrate the long-term cumulative effects of climate change on MP pollution.

## 3 Interactions with biogeochemical cycles

Microplastics significantly influence carbon cycling and the biological pump in marine ecosystems. They are integrated into the particulate organic carbon (POC) pool, affecting the efficiency of carbon export to the deep sea (Wu et al., 2025). Moreover, MPs are transported to the deep ocean by adhering to sinking organic matter, often called “marine plastic snow” (Galgani et al., 2022). Marine snow (MS), composed of phytoplankton, detritus, fecal pellets, and exopolymeric substances (EPS), plays a crucial role in the biological carbon pump by transporting organic material and nutrients to deeper layers (Baumas and Bizic, 2024). Recent studies estimated that 3–11 million metric tonnes of plastic pollution reside on the ocean floor as of 2020, highlighting the role of MS in driving plastic flux across different ocean layers and into deep-sea sediments (Zhu et al., 2024; Ziervogel et al., 2024; Wu et al., 2025). A study by Galgani et al. (2022) showed that carbon contained in MPs may contribute up to 3.8% of the downward flux of particulate organic carbon, affecting the efficiency of carbon export from the surface to the deep ocean. In the North Pacific Subtropical Gyre, MPs were found to comprise a substantial part of the POC pool, with concentrations reaching 334 particles/m^3^ (Zhao et al., 2023). This shift could have long-term consequences for carbon cycling and marine ecosystems, particularly as MPs accumulate in sediments.

Climate-driven changes significantly impact the sorption of contaminants, including MPs, in freshwater ecosystems. These changes alter the dynamics of MP pollution, affecting their concentration, distribution, and interaction with other contaminants (Khant et al., 2024). The increasing frequency of extreme weather events and prolonged droughts, driven by climate change, exacerbates MP pollution by altering river hydro-sedimentary regimes and increasing local concentrations, which pose threats to aquatic organisms (Joo, 2026). A study using regression random forest models projected that a 10 °C increase in temperature could enhance plastic degradation, resulting in a significant increase in MP concentrations (Chang et al., 2024). Microplastics can act as vectors for persistent organic pollutants (POPs) and metals, with sorption influenced by environmental factors like pH, salinity, and temperature (Chang et al., 2024; Joo, 2026). The bioaccessibility of contaminants sorbed to MPs varies widely, affecting the potential risks of exposure to aquatic organisms and humans. While the current understanding of MP interactions in freshwater systems is limited, it is clear that climate change exacerbates these challenges. Therefore, future research should explore the long-term, synergistic, and cumulative risks posed by MP under changing climate scenarios.

## 4 Biological responses and toxicological synergies

Microplastics have several properties, including polymer type, size, and shape, which can influence their adverse effects on aquatic organisms. They were detected in 75% of the mussels (*Perna*) sampled in Santos estuary and 77% of *E. japonicus* sampled in Tokyo Bay (Santana et al., 2016). Moreover, MPs were found to block the digestive tract and induce internal damage, leading to disruption of food ingestion (Figure 1) (Andrady, 2017; D'Avignon et al., 2023). Previous studies demonstrated that MPs induced oxidative stress and inhibited the growth and reproduction of marine organisms (Figure 1) (Lei et al., 2018; Du et al., 2020; Lyu et al., 2021). Moreover, Karami et al. (2016) reported that 500 μg/L of low-density polyethylene (LDPE) could downregulate the transcription of gonadotropin-releasing hormone (*GnRH*) in the hypothalamus of *Clarias gariepinus*, and vitellogenin (*Vtg*) and choriogenin (*Chg*) in the liver of *O. latipes*. The downregulation of *GnRH*, *Vtg*, and *Chg* genes suggests that MP might cause reproductive disruption (Karami et al., 2016; Sugierski et al., 2025).

Thermal stress exacerbates MP toxicity depending on the species and concentrations, leading to synergistic effects that result in oxidative stress, increased mortality, histopathological alterations, reduced growth, and reproductive impairment in aquatic organisms (Table 1). Studies on *Acropora cervicornis* (0.00125 mg/l MP) and *Pocillopora damicornis* (10 mg/L) reveal that exposure under warming conditions (25 °C and 30 °C) leads to reduced skeletal growth and disrupted symbiotic relationships, evidenced by lower chlorophyll concentrations and increased expression of stress response genes (Isa et al., 2024; Sugierski et al., 2025). High temperatures increase ingestion rates of MPs due to augmented metabolism that requires more food, causing enhanced oxidative stress, irregular metabolism, and severe physiological damage in aquatic organisms such as *Daphnia* (30 °C) and *juvenile* fish (24 °C) (Hoffschröer et al., 2021; Lyu et al., 2021). In addition, high water temperatures above 20 °C–30 °C make MPs more lethal, with toxicity becoming more prominent as temperatures rise (Junaid et al., 2023). Jaikumar et al. (2018) reported an increase in the lethal toxicity of 10^7^ particles/ml 1–5 µm primary MPs in *Daphnia magna* under elevated temperatures (26 °C) compared to 18 °C and 22 °C temperature, with *Ceriodaphnia dubia* showing no significant difference across different temperatures. Hoffschröer et al. (2021) also reported an increased bioconcentration of MPs in *D. magna* and *Daphnia pulex* at elevated temperatures (24 °C or 30 °C) under acute exposures, with no evidence of toxicity effect. It is evident that the short-term effect of microplastics may be negligible, whereas a long-term exposure to increased temperature may elevate metabolism, which increases food consumption, hence, an increased rate of MPs uptake due to mistaking them for food. Increased MP consumption allows build-up, which induces oxidative stress in *D. magna*, potentially leading to lethal toxicity (Lyu et al., 2021).

**TABLE 1:** The response of aquatic biota to different microplastics and thermal stressors.

Biota	Species	Microplastic interacting with a thermal stressor	Effect	References
Fish	*Harpagifer bispinis*	200 mg/L Polyvinyl chloride (PVC) at 12 °C	Oxidative stress	Nualart et al. (2025)
Fish	*Oreochromis niloticus*	10 mg/L Polyamide (PA) at 33 °C and 36 °C	Gills and intestinal histopathology	Hasan et al. (2022)
Tadpoles	*Lithobates catesbeiana*	1 mg/L Polystyrene at 32 °C	Oxidative stress and inflammatory response	Wang et al. (2025)
Clam	*Ruditapes philippinarum*	10 mg/L Polyethylene terephthalate at 23 °C	Survival	Song et al. (2025)
Oyster	*Crassostrea gasar*	0.1 mg/L polystyrene at 28 °C	Oxidative stress	Ferreiro et al. (2025)
Freshwater shrimp Sowbug	*Gammarus pulex* *Asellus aquaticus*	23 MP/mL High-density polyethylene at 10, 15, and 20 °C 23 MP/mL High-density polyethylene at 10, 15, and 20 °C	No effect No effect	Marchant et al. (2025) Marchant et al. (2025)
Trichoptera	*Sericostoma pyrenaicum*	180 MP/mL Polystyrene (PS) at 31.4 °C	Survival and food consumption	Firmino et al. (2023)
Daphnids	*Daphnia pulex*	10 mg/L Polyester (PES) at 20 °C and 24 °C	Survival and reproduction	Klasios et al. (2024)
Cyanobacteria	*Anabaena variabilis*	0.4 mg/mL Polyethylene (HDPE) at 25 °C–32.5 °C)	Growth	Gopalakrishnan and Kashian (2023)
Green algae	*Pseudokirchneriella* sp	5 mg/L Polymethylmethacrylate (PMMA) at 25 °C	Growth and fatty acid content	Yaripour et al. (2025)
Green algae	*Dunaliella tertiolecta*	0.05 mg/L Polystyrene (PS) at 30 °C	Growth and productivity	Vukosav et al. (2026)

Nevertheless, Marchant et al. (2025) observed an inconclusive trend on the influence of high-density polyethylene (HDPE) on *Gammarus pulex* and *Asellus aquaticus* with increasing temperature (10, 15 °C and 20 °C), whereas an antagonistic effect of fluorescent polyethylene and thermal stress was observed on the post-exposure performance of *Symphysodon aequifasciatus* (Wen et al., 2018). Moreover, Weber et al. (2020) observed no significant difference on oxidative stress, energy reserves, and activity of mussel (*Dreissena polymorpha*) after exposure to different concentrations of polystyrene (0.0064, 0.16, four or 100 MP/L) at 14, 23 °C and 27 °C. It is clear that more studies still need to be done on the combined effect of the two stressors due to their inconclusive response due to their broader ecological repercussions as well as socioeconomic implications.

For producers in aquatic environments, Cousins et al. (2025) reported no significant difference for phytoplankton biomass exposed to polyethylene at 6 °C and 18 °C. Moreover, MPs and temperature increase were found to enhance carbon and nitrogen storage in algae (Sun et al., 2023). In contrast, MPs leachates due to UV radiation reduced algal biomass, cell growth, and photosynthetic activities on microalgae (Rummel et al., 2022). While the combined effects of thermal stress and MP exposure pose serious threats to aquatic life, it is essential to consider that some species may exhibit resilience under certain conditions or adaptive mechanisms that could mitigate the impacts of MPs and associated pathogens. Thus, further research is necessary to understand the long-term implications of these stressors in aquatic ecosystems and the potential for recovery after system restoration. Moreover, studies to elucidate the underlying mechanisms, particularly considering global environmental changes and their implications in the physiology and the dynamics of ecosystem functioning.

## 5 Policy perspectives

Microplastic pollution in freshwater systems has only gained traction over the past few decades, and its toxicity effects on the environment are still debatable. Despite overwhelming evidence proving the effect of microplastics on the environment (Aljaibachi et al., 2020; Assas et al., 2020; Boyero et al., 2020; Amponsah et al., 2024), there are still uncertainties pertaining to their ecological risks, particularly when co-occurring with other stressors. Moreover, their recognition as pollutants in developed countries is already reflected in their legislation and policies; however, there are still some uncertainties regarding the applicability of these frameworks beyond country borders. Moreover, climate change addressing policies and conventions have a footprint in every continent; nevertheless, they are still talking to sole stressors, with little covered regarding the interactive phenomenon. Despite climate change showing a considerable association with plastics, the integration of plastic pollution into its policy frameworks is yet to be explored.

Sunil et al. (2024) argued that although microplastics are showing an association with climate change, they in turn contribute to greenhouse gas emissions and also affect the complex microbial communities driving such emissions. Numerous countries have legislation restricting the single-use of plastics (Clayton et al., 2021; Malkin and Green, 2025). The Global Plastics Treaty has been signed by 175 nations to deal with microplastic pollution through a comprehensive approach, from its production to disposal (Ammendolia et al., 2025). OECD (2021) proposed a source-directed policy approach, use-oriented policy approach, end-of-pipe policy approach and end-of-life policy approach to make improvements in the mitigation of microplastic pollution. The Australian government introduced the National Plastic Plan in 2021 to address microplastics from the sources, enhancing recycling, and promoting research and data sharing (DAWE, 2021). Moreover, the producer responsibility principles and the implementation of zero waste policies are recommended to minimize plastic waste in the environment (Alpizar et al., 2020). Nevertheless, there is no international legislation that comprehensively addresses plastic pollution due to its untraceable multiple sources and little effort to provide alternative solutions. Replacing synthetic plastics with biodegradable ones as a strategy to address microplastics pollution is gaining momentum (OECD, 2021); nevertheless, the impact is still negligible due to limited participation by global communities. Microplastics have been found in remote areas such as the top of the mountains, Arctic and Antarctic regions, groundwater, caves, and remote rivers. Therefore, addressing microplastics pollution requires global collaborative efforts with regard to policy direction and the involvement of citizen science to integrate end-users in solution seeking.

## 6 Conclusion and future research directions

Pollutants in the environment occur in a mixture, whereas most research explore main rather than the combined effects. Considerable studies on emerging contaminants such as microplastics showed overwhelming evidence of their occurrence and toxicity pathways, neglecting the role of other co-occurring stressors influencing the toxicity dynamics. Temperature plays a crucial role in the leaching and the toxicity kinetics of microplastics, and also increases the rate of greenhouse gas emissions. Moreover, an extreme hydrologic regime due to climate change was found to accelerate microplastic build-up in aquatic environments. This review presents evidence that stressors in aquatic ecosystems are not acting in silo and multiple stressors still produce cascading and non-linear effects, resulting in a poor understanding of the interactive effect by stressors such as microplastics, climate change, and chemical pollution. For future studies, a shift towards holistic and system-level modeling of interactions between microplastics, climate change, and chemical pollution are recommended. Moreover, studies employing predictive models to project possible synergistic effects of different contaminants are still lacking. The long term response of freshwater ecosystems to co-occurring multiple stressors is poorly explored; therefore, comprehensive monitoring from co-stressors to coupled Earth system processes is recommended to enhance understanding of the long term trajectory.
